# Docetaxel and epirubicin compared with docetaxel and prednisone in advanced castrate-resistant prostate cancer: a randomised phase II study

**DOI:** 10.1038/bjc.2011.5

**Published:** 2011-02-01

**Authors:** R Petrioli, A Pascucci, R Conca, G Chiriacò, E Francini, G Bargagli, A I Fiaschi, A Manganelli, G De Rubertis, G Barbanti, R Ponchietti, G Francini

**Affiliations:** 1 Medical Oncology Unit, University of Siena, Viale Bracci 11, 53100, Siena, Italy; 2 Pharmacology Unit, University of Siena, Viale Bracci 11, 53100 Siena, Italy; 3 Department of Urologic Surgery, University of Siena, Viale Bracci 11, 53100 Siena, Italy; 4 Genitourinary Unit, University of Siena, Viale Bracci 11, 53100 Siena, Italy

**Keywords:** castrate resistant, prostate cancer, epirubicin, docetaxel, bone metastases, prostate-specific antigen

## Abstract

**Background:**

This randomised phase II study compared the activity and safety of the combination docetaxel (D)/epirubicin (EPI) with the conventional treatment D/prednisone (P) in advanced castrate-resistant prostate cancer (CRPC) patients.

**Materials and methods:**

Patients were randomly assigned to D 30 mg m^−2^ as intravenous infusion (i.v.) and EPI 30 mg m^−2^ i.v. every week (D/EPI arm), or D 70 mg m^−2^ i.v. every 3 weeks and oral P 5 mg twice daily (D/P arm). Chemotherapy was administered until disease progression or unacceptable toxicity.

**Results:**

A total of 72 patients were enrolled in the study and randomly assigned to treatment: 37 to D/EPI and 35 to D/P. The median progression-free survival (PFS) was 11.1 months (95% CI 9.2–12.6 months) in the D/EPI arm and 7.7 months (95% CI 5.7–9.4 months) in the D/P arm (*P*=0.0002). The median survival was 27.3 months (95% CI 22.1–30.8 months) in the D/EPI arm and 19.8 months (95% CI 14.4–24.8 months) in the D/P arm (*P*=0.003). Both regimens were generally well tolerated.

**Conclusion:**

The treatment of advanced CRPC with weekly D combined with weekly EPI was feasible and tolerable, and led to superior PFS than the treatment with 3-weekly D and oral P.

The results of two large randomised trials have provided substantial support in favour of the role of chemotherapy in the treatment of castrate-resistant prostate cancer (CRPC) by demonstrating that docetaxel (D) and prednisone (P) improve survival in comparison with older regimens, and significantly improve the quality of life (Petrylak *et al*, 2004; Tannock *et al*, 2004).

Although D/P combination has become the first-line standard of care for advanced CRPC, PSA responses rarely exceed 50% and median survival is less than 20 months. The use of chemotherapy in CRPC therefore remains a subject of active clinical investigation, and the mild toxicity of D makes it an attractive treatment option for the development of combination regimens.

Docetaxel is usually administered at a dose of 70–75 mg m^−2^ every 3 weeks or on a weekly basis at a dose of 30–36 mg m^−2^. However, according to the TAX327 trial, weekly D was not equally effective when compared with D given at 3-week intervals in the treatment of advanced CRPC, and furthermore, weekly D was not superior to mitoxantrone (Tannock *et al*, 2004). The toxicity profiles of the weekly and 3-weekly treatment are distinctly different (Engels and Verweij, 2005). The acute toxicity of weekly D (particularly myelosuppression) is mild and never dose limiting, which is why this schedule is proposed above all for patients with poor performance status (PS), decreased haematological reserve or multiple co-morbidities, and for elderly patients (Beer *et al*, 2001; Petrioli *et al*, 2003).

Among other chemotherapeutic agents, doxorubicin has shown some activity in CRPC, and we have previously described the satisfactory effectiveness of the doxorubicin analogue epirubicin (EPI) that, at equipotent doses, is associated with quantitatively less severe toxicities than its parent compound (Francini *et al*, 1993; Brausi *et al*, 1995; Petrioli *et al*, 2002). More recently, we reported, in a phase II study, the activity (68.4% PSA response and 72.7% palliative response) and tolerability of the combination of weekly D and weekly EPI in advanced CRPC patients (Petrioli *et al*, 2007). The promising results observed during the course of that experience provided the rationale to compare the activity of the combination D/EPI with the conventional treatment D/P in terms of progression-free survival (PFS) in advanced CRPC patients.

## Patients and Methods

### Eligibility criteria

The study involved patients with histologically confirmed, measurable, or evaluable advanced prostatic adenocarcinoma progressing during hormonal therapy. They were admitted to the chemotherapy protocol, provided that they met at least one of the following criteria: a positive bone scan and a ⩾25% increase in PSA (PSA higher than 5 ng ml^−1^) in comparison with baseline on two successive occasions separated by at least 2 weeks for patients without measurable disease; new metastatic lesions revealed by a bone scan; and a 25% increase in a bidimensionally measurable tumour mass. The patients treated with LH-RH agonists had to continue their primary androgen ablation therapy, and were required to have serum testosterone levels of <50 ng ml^−1^ before study entry. Anti-androgen agents had to be stopped 4–6 weeks before the use of chemotherapy to allow the withdrawal to become effective. All of the patients had to have an Eastern Cooperative Oncology Group PS of ⩽2 and adequate haematological (leukocytes ⩾3000 mm^−3^; haemoglobin ⩾10 g dl^−1^; and platelets ⩾100 000 mm^−3^), renal (serum creatinine ⩽2.0 mg dl^−1^), and hepatic functions (serum bilirubin ⩽2.0 mg dl^−1^). The exclusion criteria were previous chemotherapy, congestive heart failure, a recent myocardial infarction, or any other previous malignant disease except basal cell carcinoma of the skin. All of the patients gave their informed consent, and the protocol was approved by the Ethics Review Board of Siena University.

### Treatment plan

In D/EPI arm, the treatment consisted of D 30 mg m^−2^ as a 30-min intravenous infusion (i.v.) and a bolus of EPI 30 mg m^−2^ diluted with 100 ml of saline solution, based on a schedule of 6 consecutive weekly administrations, followed by a 2-week rest interval. In D/P arm, treatment consisted of D 70 mg m^−2^ intravenously every 3 weeks and oral P 5 mg twice daily. Given that elderly patients, patients with co-morbidities, and/or patients with poor PS are not expected to tolerate the full-dose 3-weekly regimen (Engels and Verweij, 2005), a slightly lower dose of D (70 mg m^−2^) instead of the conventional 75 mg m^−2^ was chosen for the current trial.

Cycles were administered, if serum WBC levels were >3000 mm^−3^, granulocytes >1500 mm^−3^, and platelets >100 000 mm^−3^. Pre-medication consisted of dexamethasone 8 mg orally 12 h before, at the time of, and 12 h after D administration. Ondansetron 8 mg was administered at the beginning of each treatment cycle as antiemetic medication. The patients continued to take analgesic medication at doses adjusted to provide optimal pain control. All patients with bone metastases received bisphosphonates (zoledronic acid 4 mg i.v. every 4 weeks).

The chemotherapy was administered until disease progression or unacceptable toxicity, and for a maximum of 30 weekly cycles (EPI for a maximum of 24 cycles) in the D/EPI arm and for a maximum of 10 three-weekly cycles in the D/P arm. The maximum planned cumulative dose of EPI was 720 mg m^−2^.

### Response assessments

Serum PSA was measured every 3 weeks, and PSA criteria for response and progression were based on the PSA Working Group (PWG) consensus criteria (Bubley *et al*, 1999): a PSA response was defined as a reduction from baseline of at least 50% for at least 3 weeks (instead of 4 weeks in the PWG criteria), whereas PSA progression was defined as an increase from nadir of at least 25% in patients without biochemical response or ⩾50% PSA increase in patients with previous biochemical response. Blood and platelet counts, and a comprehensive screening profile were performed at baseline and every 3 weeks. The patients in the D/EPI arm underwent a weekly complete blood cell count before chemotherapy. The baseline imaging studies included abdominal and pelvic CT or magnetic resonance imaging, a bone scan, and chest radiography. All measurable disease (not bone lesions) was re-evaluated at 8-week intervals using the Response Evaluation Criteria in Solid Tumors (Therasse *et al*, 2000), and a radionuclide bone scan was repeated every 6 months.

All of the patients underwent a baseline ECG, and their left ventricular ejection fraction (LVEF) was measured by means of a multiple gated acquisition scan or echocardiography at baseline and every 12 weeks; further cardiac examinations were performed as indicated. A major decrease in LVEF was defined as an absolute decrease to at least 15% below the lower normal limit.

A radionuclide bone scan was performed at baseline and repeated every 6 months. Bone markers were measured at baseline and after 3 months of treatment: the bone resorption marker s-CTX (serum C-telopeptide of collagen type I) was evaluated by an ELISA method (Serum Cross Laps ELISA, Nordic Bioscience Diagnostics, Herlev, Denmark) and the bone formation marker B-ALP (bone-alkaline phosphatase) by a radioimmunometric method (B-ALP Tandem-R Ostase, Hybritech, Merceville, NJ, USA). Bone disease progression was defined as the appearance of any new bone lesion or the progression of existing bone metastases.

Pain symptomatology was measured at baseline and then every 6 weeks by the McGill Melzack Pain Questionnaire, and pain response was defined as a 2-point reduction in the 6-point present pain intensity scale (or the complete disappearance of pain, if the initial score was 1+) (Melzack, 1975). These results had to be maintained at two consecutive evaluations made at least 3 weeks apart and without any increase in analgesic consumption. The patients were asked to classify the average pain level during the previous 24 h: we used a translated form of the McGill Melzack Questionnaire to which the ‘reconstruction-based methodology’ has been applied (De Benedittis *et al*, 1988). Analgesic consumption was based on the average daily quantities taken by the patient during the previous week and assigned oral morphine equivalents before analysis (McCormack *et al*, 1992).

To evaluate the impact of treatment on quality of life, all patients were asked to complete the EORTC QLQ-C30 questionnaire before the start of treatment and thereafter every 6 weeks until they went off the trial (Aaronson *et al*, 1993). Analyses were restricted to changes at 12 and 18 weeks of global quality of life in which physical function, pain, fatigue, and nausea/vomiting were considered. Clinical significant changes required changes of ⩾10% points (Osoba *et al*, 1998).

### Treatment-related adverse events

Toxicity was defined using the National Cancer Institute (NCI) Common Toxicity Criteria, version 3.0 (National Cancer Institute, Bethesda, MD, USA). The treatment was delayed at the first occurrence of grade II haematological toxicity, and administered at the same dose after it returned to grade I or better. In the case of grade III or IV toxicity, the treatment was interrupted and a maximum of 3 weeks were allowed for recovery, after which the patients were withdrawn from the study. In the case of a second episode of grade III or IV toxicity in the same patient, treatment was resumed after recovery and the subsequent administrations of each drug were reduced to 20 mg m^−2^. A prophylactic use of haematopoietic growth factors was not applied. Chemotherapy was discontinued, if the ejection fraction decreased below the institutional lower limit of normal and declined by ⩾15%.

### Statistical analysis

The primary end point was the comparison of PFS between groups in the per-protocol population. The PFS was calculated as the time from the first chemotherapy infusion to disease progression or death. Previous trials investigating the conventional D/P treatment indicate that ∼60% of patients are progression free 6 months after treatment onset. The hypothesis for the current study was that, using the combined D/EPI schedule, at least 75% of patients would be progression free after 6 months from the start of chemotherapy. It was calculated that 32 evaluable patients per arm would have to be recruited to yield a 90% probability to correctly select the best treatment when it is superior by absolute difference of 15% in response rate (Simon *et al*, 1985). It was planned to enrol at least 70 patients in the expectation of 10–15% of unevaluable cases. Patients were randomised by using a computer-generated random list, and there were no stratification factors.

Secondary end points were safety, PSA response, duration of response (RD), changes in bone markers, quality of life, and overall survival (OS). The PFS, RD, and OS were determined using the Kaplan–Meier method to provide the median value and 95% CI, and treatment groups were compared using the log-rank test. Comparison of median percentage change from baseline of bone markers during the observation period between the two groups was analysed using the Mann–Whitney test. All tests were two –sided, with a significance level of 0.05. Baseline clinical characteristics, response rates, and adverse events were compared using *χ*^2^ statistics. All data were analysed by MedCalc software (MedCalc Statistical Software, Mariakerke, Belgium).

## Results

### Patient characteristics

Between October 2005 to January 2010, 72 advanced CRPC patients were enrolled in the study and randomly assigned to treatment: 37 to D/EPI and 35 to D/P. The recruitment period was 4.3 years, and this enough extensive time was mainly because of the monocentric characteristic of the study. The median age was 72 years (range 53–82 years) in the D/EPI arm and 70 years in the D/P arm (59–83 years). A total of 32 patients in the D/EPI arm and 29 patients in the D/P arm had bone metastases: 12 patients in the D/EPI arm and 13 patients in the D/P arm had measurable disease. Median baseline serum PSA was 82 ng ml^−1^ (range 14–182 ng ml^−1^) in the D/EPI arm and 66 ng ml^−1^ (range 11–253 ng ml^−1^) in the D/P arm (Table 1).

### Treatment

A total of 893 weekly cycles of D/EPI (median 25 cycles, range 1–30) and a total of 253 three-weekly cycles of D/P (median 7 cycles, range 3–10) were administered in the evaluable per-protocol population.

### Efficacy

**Figure 1:**
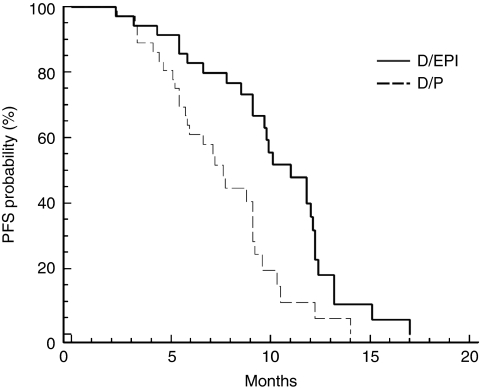
Estimated PFS for advanced CRPC patients randomly assigned to D/EPI (—) or D/P (– – – –) treatment.

The median duration of follow-up was 28.5 months (range 0.5–38.5 months): one patient in the D/EPI arm received only one treatment cycle for chemotherapy-unrelated reasons and was lost to follow-up; two patients in the D/EPI arm and one patient in the D/P arm were lost to follow-up after the first 6 months. All patients were included in the overall analysis (intent-to-treat). The efficacy results are summarised in Table 2. After 6 months from the onset of treatment, 83.7% of patients in the D/EPI arm and 57.1% of patients in the D/P arm were free from progression (*P*=0.02). The PFS differed significantly between the two treatment arms: median of 11.1 months (95% CI 9.2–12.6 months) in the D/EPI arm *vs* 7.7 months (95% CI 5.7–9.4 months) in the D/P arm (*P*=0.0002) (Figure 1).

A total of 32 patients (86.4%) in the D/EPI arm and 30 (85.7%) in the D/P arm received a second-line chemotherapy: 23 patients in the D/EPI arm (62.1%) and 16 patients in the D/P arm (45.7%) were retreated with D after completion of first-line chemotherapy. As salvage treatment, 24 patients in the D/EPI arm and 18 patients in the D/P arm were enrolled in an ongoing trial with a treatment consisting of weekly D and the anti-angiogenic agent bevacizumab (Bev).

**Figure 2:**
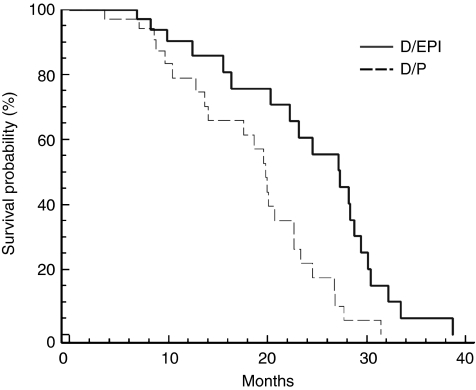
Estimated OS for advanced CRPC patients randomly assigned to D/EPI (—) or D/P (– – – –) treatment.

As of 31 August 2010, 21 patients in the D/EPI arm and 26 patients in the D/P arm had died: the median survival was 27.3 months (range 7.2–38.4 months; 95% CI 22.1–30.8 months) with D/EPI *vs* 19.8 months (range 3.8–31.6 months; 95% CI 14.4–24.8 months) with D/P (*P*=0.003) (Figure 2).

A confirmed >50% decrease in PSA was achieved in 28 patients (75.6% 95% CI 59.8–86.6%) in the D/EPI arm and in 19 patients (54.2% 95% CI 38.1–69.5%) in the D/P arm (*P*=0.09). The PSA levels decreased >75% in 22 patients in the D/EPI arm (PSA returned <4 ng ml^−1^ in 8 patients) and in 7 patients in the D/P arm (PSA returned <4 ng ml^−1^ in 3 patients). A total of 26 of the 28 PSA responses in the D/EPI arm and 14 of the 19 PSA responses in the D/P arm were observed within the first 8 weeks of treatment. Five patients (13.5%) in the D/EPI arm and 11 (31.4%) in the D/P arm had stable PSA levels for at least 3 months. A partial response on measurable disease was achieved in seven patients (58.3%) in the D/EPI arm and in five patients (38.4%) in the D/P arm.

In subjects who were symptomatic at baseline, pain was reduced in 24 patients (72.7% 95% CI 55.6–84.9%) in the D/EPI arm and in 13 (43.3% 95% CI 27.3–60.9%) in the D/P arm (*P*=0.02). The median duration of palliative response was 10.6 months in the D/EPI arm and 5.9 months in the D/P arm (*P*=0.003).

Bone scan, which could be repeated after 6 months (range 6–8 months) in 31 patients in the D/EPI and in 27 patients in the D/P arm, revealed two or more new lesions compared with scan at trial entry in one patient in the D/EPI arm and in 4 patients the in D/P arm.

Of 32 patients with bone metastases in the D/EPI arm, 25 (78.1%) and 21 (65.6%) had baseline s-CTX and B-ALP above the normal range, respectively. Of 29 patients with bone metastases in the D/P arm, 21 (72.4%) and 18 (62.0%) had baseline s-CTX and B-ALP above the normal range, respectively. At 3 months of treatment, the median s-CTX reduction was 71% (95% CI 58.3–71.4%) below baseline in the D/EPI group compared with 66% (95% CI 56.2–75.8%) in the D/P arm (*P*=0.21). At 3 months, both chemotherapy regimens similarly decreased the bone marker B-ALP (D/EPI 62.4% 95% CI 47.3–74.4% and D/P 59.1% 95% CI 42.8–71.7%).

### Quality of life

Baseline QLQ-C30 data were available for 67 patients (34 D/EPI and 33 D/P). Compliance with QL assessment decreased to 63 patients (87.5%) after 12 weeks and 56 (77.7%) after 18 weeks. At 12 and 18 weeks, global quality of life improved in 54.5 and 44.8% of patients in the D/EPI arm, and in 30.0 and 23.3% of patients in the D/P arm, respectively. There was a trend (*P*⩽0.1) towards a better improvement in physical function and pain subscales in the D/EPI arm compared with the D/P arm: nonetheless, the difference in these quality of life parameters between the two treatment groups did not reach the statistical significance. At 12 and 18 weeks, global quality of life worsened in 6.0 and 13.3% of patients in the D/EPI arm and in 9.6 and 16.0% of patients in the D/P arm, respectively.

### Toxicity

Both regimens were generally well tolerated, and no unexpected toxic effects were observed (Table 3). The median received cumulative dose of 710 mg m^−2^ (range 30–750 mg m^−2^) for D and 630 mg m^−2^ (range 30–720 mg m^−2^) for EPI in the D/EPI arm, and 560 mg m^−2^ (range 190–700 mg m^−2^) for 3-weekly D in the D/P arm.

No grade 4 toxicity or congestive heart failure was observed, and 99% of the cycles in the D/EPI arm and 97% of the cycles in the D/P arm were administered on an outpatient basis. The most frequent side effects were neutropaenia, anaemia, thrombocytopaenia, and fatigue, which were grade I or II in most cases. Grade III/IV neutropaenia occurred in 18.9% of patients in the D/EPI arm and in 28.5% of patients in the D/P arm. The D/EPI was associated with a trend for more grade 2–3 fatigue, nail changes, and dry eye. Fatigue achieved grade 3 in three patients in the D/EPI arm and in two patients in the D/P arm; the treatment was resumed in all these patients after a 2-week interval.

The administration of at least one treatment cycle was delayed by 1 week in 28 patients in the D/EPI arm and in 31 patients in the D/P arm: the reasons for the delays were haematological in 24 (85.7%) and 28 cases (90.3%), respectively, and non-hematological in 4 (14.3%) and 3 patients (9.7%), respectively. The dose of D and EPI was reduced in 8 patients, and the dose of 3-weekly D in 12 patients. Two patients in the D/P arm definitely stopped the treatment because of persistent severe neutropaenia after 6 and 7 three-weekly cycles, respectively. A decrease >15% in LVEF was observed in three patients after, respectively, 24, 26, and 27 weekly cycles in the D/EPI arm, and in one patient after 8 three-weekly cycles in the D/P arm.

## Discussion

The results of this study suggest that the weekly D/EPI combination is safe and achieves encouraging results over a 3-weekly D/P regimen in terms of PFS (median: 11.1 months *vs* 7.7 months) and survival (median: 27.3 months *vs* 19.8 months) in the treatment of advanced CRPC. Moreover, although the utility of a decrease >50% in PSA as a surrogate end point for PFS and OS remains questionable (Collette *et al*, 2005); PSA response was higher and time-to-response was shorter in the D/EPI arm compared with that observed in the D/P arm. The efficacy results we observed with the weekly D/EPI combination were consistent with the best results that were reported with first-line chemotherapy in advanced CRPC patients. A recent trial reported a 90% PSA decline, 18.3 months of median PFS, and 28.2 months of median OS with Bev combined with D, P, and thalidomide (Ning *et al*, 2010). However, the use of such a multiagent treatment may not be suitable for most of CRPC patients who are elderly and with various co-morbidities. The updated data of the phase III trial CALGB 90401 reported interesting results in terms of PFS with 3-weekly D combined with Bev but no significant survival benefit over D alone, whereas a greater morbidity and mortality was associated to D/Bev (Kelly *et al*, 2010).

Although it was a monocentric study, our results seem both clinically and statistically meaningful, as this study employed a randomised design and few patients were lost to follow-up. The numbers were small, and slight imbalances with respect to the main baseline characteristics might represent a concern in the current study (Table 1). However, the Halabi nomogram suggested that patient populations were similar, with an estimated median survival of about 17 months for the D/EPI arm and about 18 months for the D/P arm (Halabi *et al*, 2003).

An intriguing point is that the median PFS and also the median survival (11.1 months and 27.3 months, respectively) in the D/EPI arm were longer than the median PFS and survival (7.4 months and 21.5 months, respectively) we observed in the phase II study with the same the D/EPI regimen (Petrioli *et al*, 2007). In our previous experience, we adopted a planned break after the first 12 weekly cycles of D/EPI in patients who responded or who had a stable disease to minimise toxicity. Thus, it seems that in advanced CRPC patients, the weekly D/EPI administered until the occurrence of progression may substantially prolong PFS compared with a stop-and-go strategy. One might also speculate that the continuative administration of D/EPI until progression can delay the time-to-emergence of resistance disease compared with a stop-and-go or an intermittent application of effective chemotherapy. However, the small sample size of the studies and the absence of a randomised comparison between different treatment strategies prevent us from drawing definite conclusions in this setting.

Another point to consider in the survival analysis is that more patients in the D/EPI arm could receive weekly D plus Bev as salvage treatment (as third- or fourth-line chemotherapy), which may offer a clinical benefit and an advantage in OS in heavily pre-treated patients (our unpublished data; Heidenreich *et al*, 2010).

A rapid, high, and long-lasting pain response was observed in the D/EPI arm after 6 weeks, with a superior statistical difference compared with the D/P arm, and was probably related to the potent rapid-onset activity of the D/EPI combination. The true contribution of EPI, particularly in reducing bone pain in symptomatic patients, has been reported by a recent review on the use of anthracyclines in prostate cancer (Petrioli *et al*, 2008).

The efficacy results correlated well with changes in the bone markers s-CTX and B-ALP in both the D/EPI and D/P arm at the third month of treatment. These findings confirm the activity of chemotherapy combined with bisphosphonates in reducing both bone resorption and bone formation markers, and suggest the role of early changes in bone markers for monitoring CRPC patients with bone metastases (Francini *et al*, 2001; Brown *et al*, 2005; Coleman *et al*, 2005).

The D/EPI arm showed a trend towards a superior improvement in a number of quality of life dimensions compared with the D/P arm, particularly pain and physical function subscales, and global quality of life. Although the integration of a prostate cancer-specific module would have been helpful, these results further suggest the palliative effect achieved by the association of D with an anthracycline in metastatic CRPC patients. It appears that patients in the D/EPI arm received three doses of corticosteroids every week as pre-medication, whereas patients in the D/P arm received them every 3 weeks. It is well established that corticosteroids can improve quality of life in metastatic CRPC patients (Tannock *et al*, 1996); however, it is unlikely that differences in corticosteroids pre-medication regimen have had a significant impact on the palliative outcomes in the D/EPI arm, as patients in the D/P arm received also continuous oral P 5 mg twice daily.

Quality of life is strictly correlated with treatment tolerability, and in this setting, the use of weekly EPI combined with weekly D was not associated with a significant increase in toxicity compared with 3-weekly D combined with P. Neutropaenia was, in the D/EPI arm, only slightly more frequent than the rate usually observed with EPI alone, whereas more grade 3/4 occurred in the D/P arm, as reported with the conventional 3-weekly D (Table 3) (Tannock *et al*, 2004). Other differences in most common adverse events were mainly related to the different schedule of D administration (i.e., weekly *vs* 3 weekly). Despite the use of an anthracycline, the D/EPI arm was associated with a low incidence of cardiotoxicity. As a matter of fact, the weekly EPI schedule was chosen because we had previously demonstrated that this drug can be administered weekly for long periods without giving rise to more than mild toxic effects (Francini *et al*, 1993; Petrioli *et al*, 2002).

As expected, the characteristic and cumulative D toxicities of fatigue, tearing, and nail disorders were mild and slightly more frequent than those reported in our previous experience with D/EPI, in which the stop-and-go strategy probably contributed to minimise their occurrence (Petrioli *et al*, 2007). In conclusion, treatment of advanced CRPC with weekly D combined with weekly EPI was feasible and tolerable, and led to encouraging results in terms of PFS over treatment with a 3-weekly D (70 mg m^−2^) regimen combined with oral P. Although the improvement in PFS may not reflect a significant improvement in OS, these results suggest that the combination of D and EPI may warrant an eventual expansion to a phase III trial on CRPC.

## Figures and Tables

**Table 1:** Main patient characteristics at baseline

	**D/EPI**	**D/P**
Enrolled patients	37	35
Median age (range), years	72 (51–82)	70 (56–83)
		
*Initial Gleason score*		
⩽7	16	17
8–10	21	18
		
*ECOG performance status*		
0	8	11
1–2	29	24
		
*Sites of metastases*		
Bone	25	22
Bone + prostate cancer	5	6
Bone + lymph nodes	2	1
Lymph nodes + prostate cancer	3	5
Lymph nodes + liver	1	0
Prostate + lung	1	1
		
Median baseline serum PSA (range), ng ml^−1^	82 (14–182)	66 (11–253)
		
*Previous treatments*		
Prostatectomy	26	24
Radiotherapy	6	8
		
*Hormone therapies*		
1	37	35
⩾2	22	18
		
*Duration of response to hormonal treatment (months)*		
Median (range)	23 (6–52)	25 (7–68)
		
*Baseline pain intensity*		
0	3	5
1	10	8
2	14	16
3	6	5
4	2	1
5	0	0
		
Median LDH, U l^−1^ (range)	278 (106–1,147)	233 (124–936)
Alkaline phosphatase, U l^−1^ (range)	131 (34–751)	147 (41–582)
Median haemoglobin, g dl^−1^ (range)	11.8 (8.5–14.2)	10.9 (8.1–13.7)
		
*Overall quality of life (EORTC)* a		
Mean score±s.d. (range)	46 ± 9.57 (28–65)	42 ± 9.24 (33–59)

Abbreviations: D=docetaxel; ECOG=Eastern Cooperative Oncology Group; EORTC=European Organization for Research and Treatment of Cancer; EPI=epirubicin; LDH=lactate dehydrogenase; P=prednisone; PSA=prostatic specific antigen.

a 0=very poor; 100=excellent.

**Table 2:** Responses to treatment

	**D/EPI**	**D/P**	***P*-value**
Enrolled patients	37	35	
PSA response (% 95% CI)	75.6 (59.8–86.6)	54.2 (38.1–69.5)	0.09
Median duration of PSA response (months; 95% CI)	12.4 (8.9–15.7)	7.5 (4.8–10.3)	0.0001
Median PFS (months; 95% CI )	11.1 (9.2–12.6)	7.7 (5.7–9.4)	0.0002
Palliative response (% 95% CI)	72.7 (55.6–84.9)	43.3 (27.3–60.9)	0.02
Median duration of palliative response (months; 95% CI)	10.6 (7.8–13.4)	5.9 (3.3–8.5)	0.003

Abbreviations: CI=confidence interval; D=docetaxel; EPI=epirubicin; P=prednisone; PFS=progression-free survival.

**Table 3:** Number of patients experiencing the most frequent treatment-related adverse events

	**D/EPI**		**D/P**	
**Toxicity**	**Grade 2 (%)**	**Grade 3–4 (%)**	**Grade 2(%)**	**Grade 3–4 (%)**
*Haematological*				
Neutropaenia	16 (43.2)	7 (18.9)	17 (48.5)	10 (28.5)
Anaemia	13 (35.1)	5 (13.5)	12 (34.2)	3 (8.5)
Thrombocytopaenia	7 (18.9)	3 (8.1)	6 (17.1)	2 (5.7)
				
*Non-haematological*				
Nausea/vomiting	6 (16.2)	3 (8.1)	12 (31.4)	9 (25.7)
Diarrhoea	4 (10.8)	1 (2.7)	5 (14.2)	1 (2.8)
Constipation	5 (13.5)	2 (5.4)	4 (11.4)	2 (5.7)
Nail changes	15 (40.5)	5 (13.5)	6 (17.9)	1 (2.8)
Dry eye/tearing	11 (29.7)	3 (8.1)	4 (11.4)	1 (2.8)
Myalgia/arthralgia	6 (16.2)	1 (2.7)	5 (14.2)	2 (5.7)
Fatigue	16 (43.2)	3 (8.1)	13 (37.1)	2 (5.7)
Sensory neuropathy	2 (5.4)	0	4 (11.4)	1 (2.8)
Peripheral oedema	3 (8.1)	1 (2.7)	7 (20.0)	2 (5.7)
Epistaxis	4 (10.8)	1 (2.7)	2 (5.7)	1 (2.8)
Dyspnoea	2 (5.4)	0	2 (5.7)	1 (2.8)

Abbreviations: D=docetaxel; EPI=epirubicin; P=prednisone.
